# Supplementary material for: Remodeling synaptic connections via engineered neuron-astrocyte interactions
Source: Nat Commun. 2026 Apr 15;17:3490. doi: 10.1038/s41467-026-71440-w (PMC13084054; doi:10.1038/s41467-026-71440-w)
Supplement: Supplementary file 1 — Supplementary Information [file 41467_2026_71440_MOESM1_ESM.pdf]

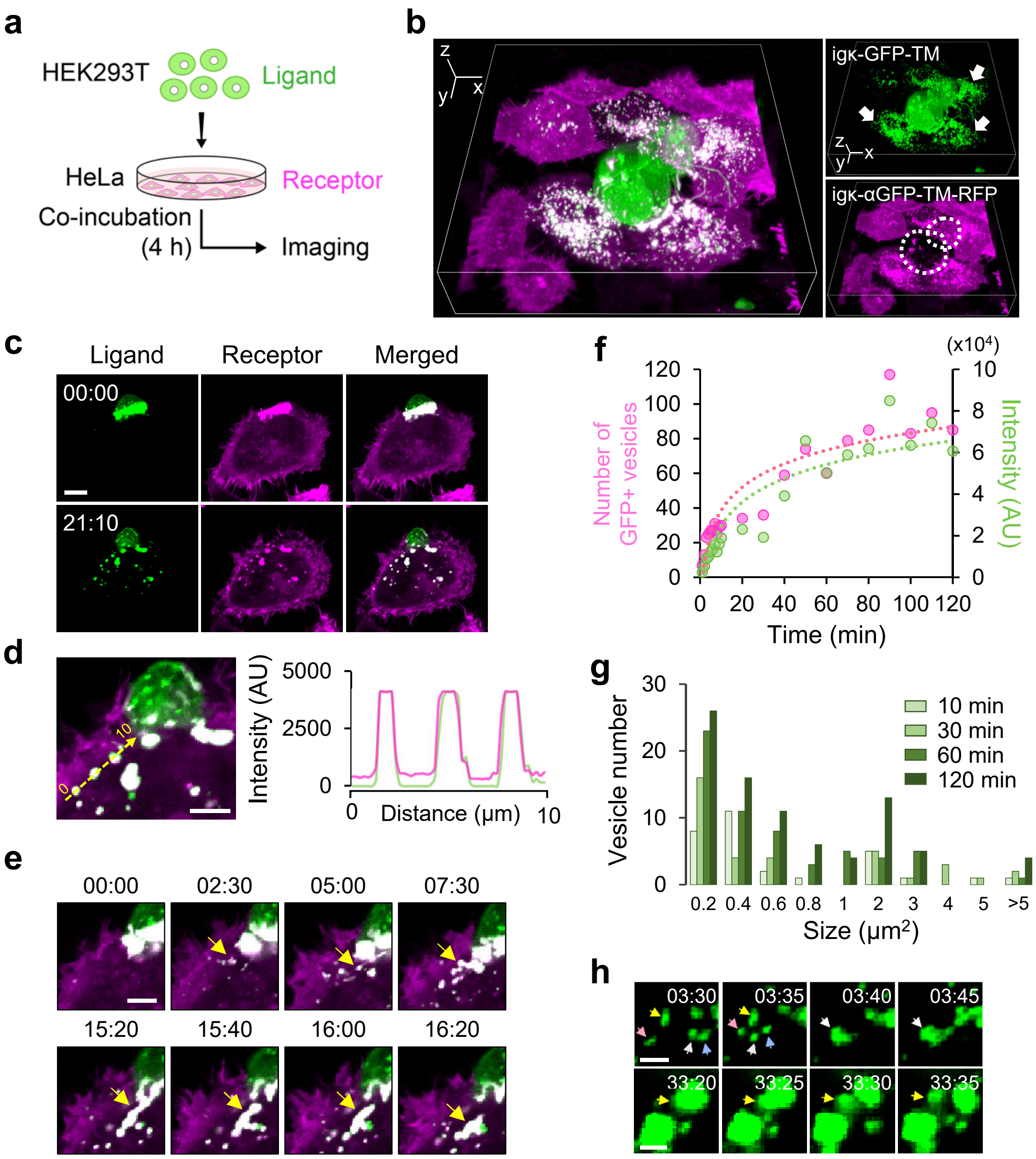

Supplementary Fig. 1. Direct cell-cell interaction-mediated SynTrogo.

a, Schematic representation of induced direct interaction between cells expressing ligand or receptor proteins. **b,** Fluorescence images demonstrating molecular transfer between ligand-expressing HEK293T cells and receptor-expressing HeLa cells 4 hours after co-incubation. Arrows in the image in the top right indicate internalized ligand molecules in HeLa cells. White dotted lines in the image in the bottom right image indicate the position of HEK293T cells. Scale bars, 10 µm. **c,** Representative fluorescence images showing cell-cell interaction and uptake of ligand molecules in HEK293T cells by receptor-expressing HeLa cells. Scale bar, 10 µm. **d,** Fluorescence image (left) and intensity profile (right) demonstrating co-localization of ligand and receptor in HeLa cell after ligand uptake. Scale bar, 5 µm. **e,** Time-lapse images of the ligand uptake process. Yellow arrows indicate ligand-containing membrane patches dissociated from a HEK293T cell. Scale bar, 5 µm. **f,** Quantification of the number of engulfed membrane patches (GFP+ vesicles) and intensity of GFP ligands in a HeLa cell. **g,** Size distribution of GFP-positive vesicles in a HeLa cell at different time points. **h,** Time-lapse images showing fusion (top) and fission (bottom) of GFP-positive vesicles after internalization. Scale bars, 2 µm. Numbers indicate minutes:seconds in **(c)**, **(e)**, and **(h)**.

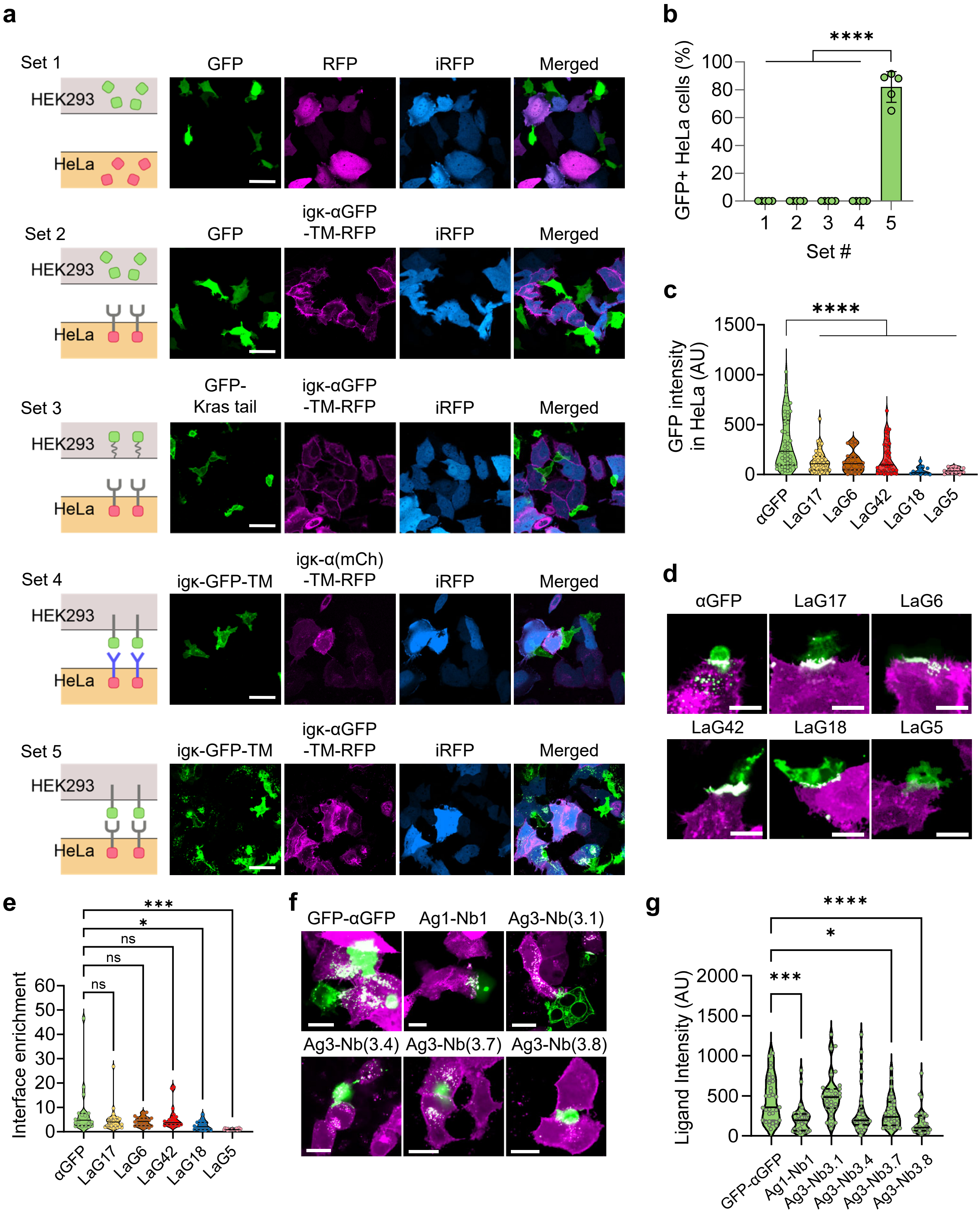

a
Set 1
HEK293
HeLa
GFP
RFP
iRFP
Merged
Set 2
igκ-αGFP
-TM-RFP
Set 3
GFP-
Kras tail
Set 4
igκ-GFP-TM
igκ-α(mCh)
-TM-RFP
Set 5
b
****
GFP+ HeLa cells (%)
100
80
60
40
20
0
1
2
3
4
5
Set #
c
****
GFP intensity
in HeLa (AU)
1500
1000
500
αGFP
LaG17
LaG6
LaG42
LaG18
LaG5
d
e
***
*
ns
Interface enrichment
60
50
40
30
10
f
GFP-αGFP
Ag1-Nb1
Ag3-Nb(3.1)
Ag3-Nb(3.4)
Ag3-Nb(3.7)
Ag3-Nb(3.8)
g
Ligand Intensity (AU)
2000
Ag1-Nb1
Ag3-Nb3.1
Ag3-Nb3.4
Ag3-Nb3.7
Ag3-Nb3.8

Supplementary Fig. 2 Characterization of SynTrogo.

a, Fluorescence images demonstrating the specificity of SynTrogo (SynT) to the interaction of ligand and receptor. Non-tagged infrared fluorescent protein (iRFP) was expressed to fill the cell volume. Scale bars, 50 μm. **b**, Percentage of GFP(+) HeLa cells under each condition described in **a**. Total number of analyzed cells were n = 108 (Set 1), 113 (Set 2), 199 (Set 3), 165 (Set 4), and 141 (Set 5) cells. **c**, GFP intensity of receptor-expressing HeLa cells under each condition using different αGFP nanobody as a part of the receptor. n = 45 (αGFP), 39 (LaG-17), 23 (LaG-6), 41 (LaG-42), 13 (LaG-18), and 11 (LaG-5) cells. **d**, Fluorescence images of each condition using different αGFP nanobody as a part of the receptor. Scale bars, 10 μm. **e**, Quantification of binding affinity using interface enrichment at contact site between receptor and ligand expressing cells. n = 19 (αGFP), 22 (LaG-17), 20 (LaG-6), 17 (LaG-42), 15 (LaG-18), and 21 (LaG-5) cells. **f**, Fluorescence images of SynT induced by various antigen-nanobody pairs. Scale bars, 20 μm. **g**, Quantification of ligand intensity in receptor-expressing cells upon the induction of SynT. n = 31 cells per group. Data are presented as mean ± s.e.m. in **b**, and as median with upper and lower quartiles (25th and 75th percentiles; dotted lines) in **c**, **e**, and **g**. Statistical significance was determined using one-way ANOVA followed by Dunnett's multiple comparison test for **b**, **c**, **e**, and **g**. *P < 0.05, ***P < 0.001, ****P < 0.0001.

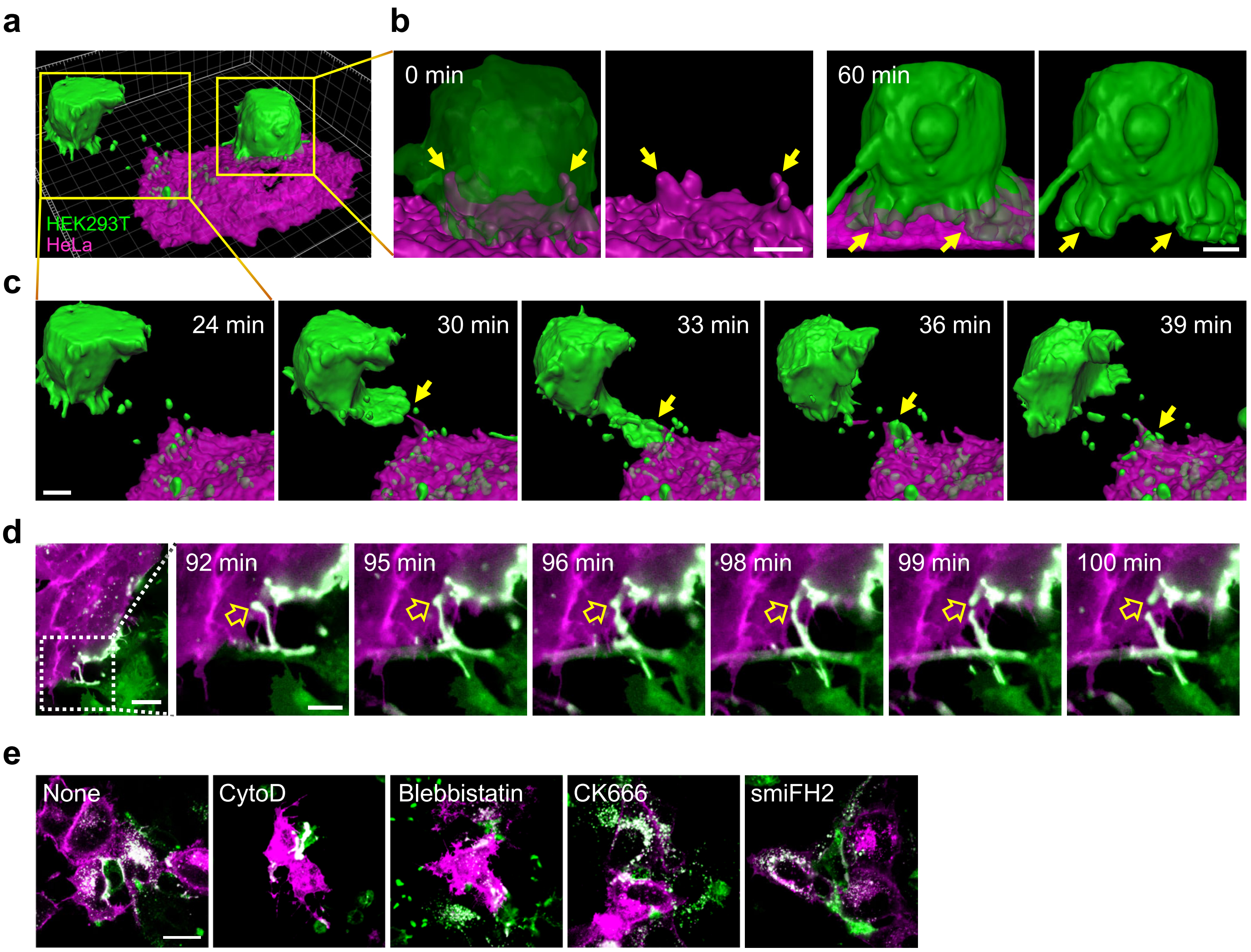

Supplementary Fig. 3. Involvement of actin polymerization in SynTrogo.

a, 3D-rendered images showing interaction between ligand- and receptor-expressing cells during SynTrogo (SynT). Z-stack images were captured every 3 minutes for 66 minutes. **b-c,** Magnified images corresponding to regions indicated by yellow boxes in **a** showing membrane structures at the interfaces of ligand- and receptor-expressing cells. Scale bars, 4 μm. **d,** Fluorescence images showing changes in membrane structure and dissociation of a membrane patch from the ligand-expressing cell during SynT. Scale bars, 10 μm (left), 5 μm (right). **e,** Fluorescence images showing the dependency of SynT on actin polymerization. Cells were treated with Cytochalasin D (CytoD, 10 μM), Blebbistatin (100 μM), CK666 (50 μM), or smiFH2 (50 μM) to block the actin polymerization, myosin-mediated actin contraction, branched actin formation, or elongated actin formation, respectively. Scale bar, 50 μm.

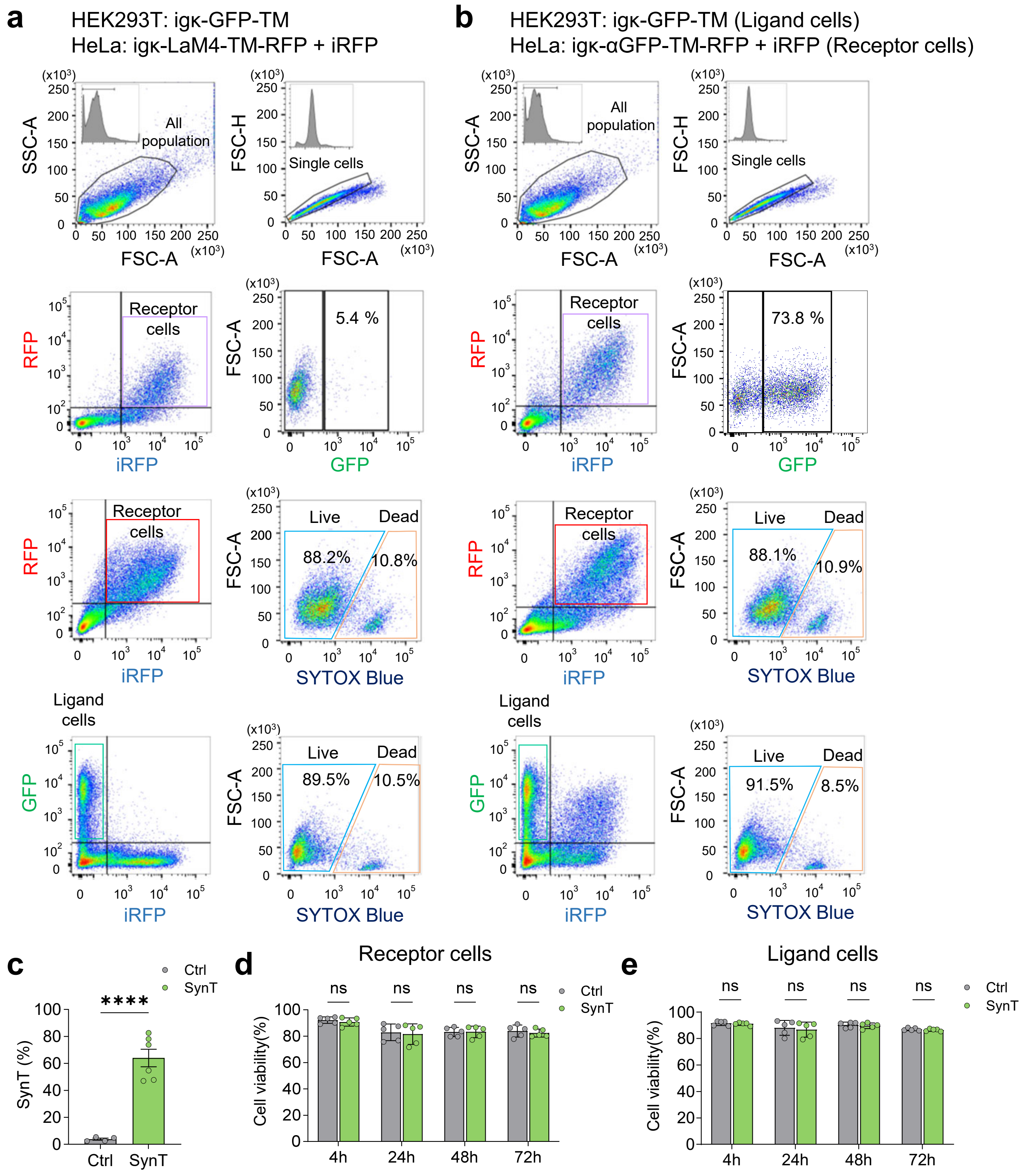
a
HEK293T: igκ-GFP-TM
HeLa: igκ-LaM4-TM-RFP + iRFP
b
HEK293T: igκ-GFP-TM (Ligand cells)
HeLa: igκ-αGFP-TM-RFP + iRFP (Receptor cells)
All population
Single cells
SSC-A
FSC-H
FSC-A
Receptor cells
Ligand cells
RFP
GFP
iRFP
FSC-A
5.4 %
73.8 %
Live
Dead
88.2%
10.8%
88.1%
10.9%
89.5%
10.5%
91.5%
8.5%
SYTOX Blue
c
d
e
Ctrl
SynT
****
SynT (%)
Receptor cells
Ligand cells
ns
Cell viability(%)
4h
24h
48h
72h

Supplementary Fig. 4. Flow cytometry analysis to examine the effect of SynTrogo on cell viability.

a-b, Representative flow cytometry plots of SynTrogo (SynT) induced by co-incubation of ligand- and receptor-expressing cells for 4 hours. **a,** mCherry nanobody (LaM4) was used as a binding module in the receptor as a control. **b,** GFP nanobody (αGFP) was used as a binding module in the receptor to induce SynT. Cells were first gated based on forward scatter (FSC-A) and side scatter (SSC-A) to exclude debris and select intact cells, followed by FSC-Height versus FSC-Area gating to exclude doublets. Receptor cells were identified as RFP+ and iRFP+ double-positive populations. The efficiency of SynT was measured as the percentage of GFP-positive receptor cells within this gated population. Cell viability was assessed using SYTOX™ Blue staining to distinguish live (SYTOX Blue–negative) from dead (SYTOX Blue–positive) cells. Gates were established using single-color compensation controls and unstained samples, and at least 10,000 events were collected per sample. **c,** Quantification of SynT efficiency. n = 6 trials. **d-e,** Following the induction of SynTrogo, (**d**) cell viability in receptor and (**e**) ligand cells was quantitatively assessed at 4, 24, 48, and 72 hours using SYTOX™ Blue staining. n = 5 trials. Data are represented as mean ± s.e.m. Statistical significance was determined using unpaired *t*-test for **c** and two-way ANOVA with Sidak's multiple comparison test for **d, e**. ****$P < 0.0001$; ns, not significant.

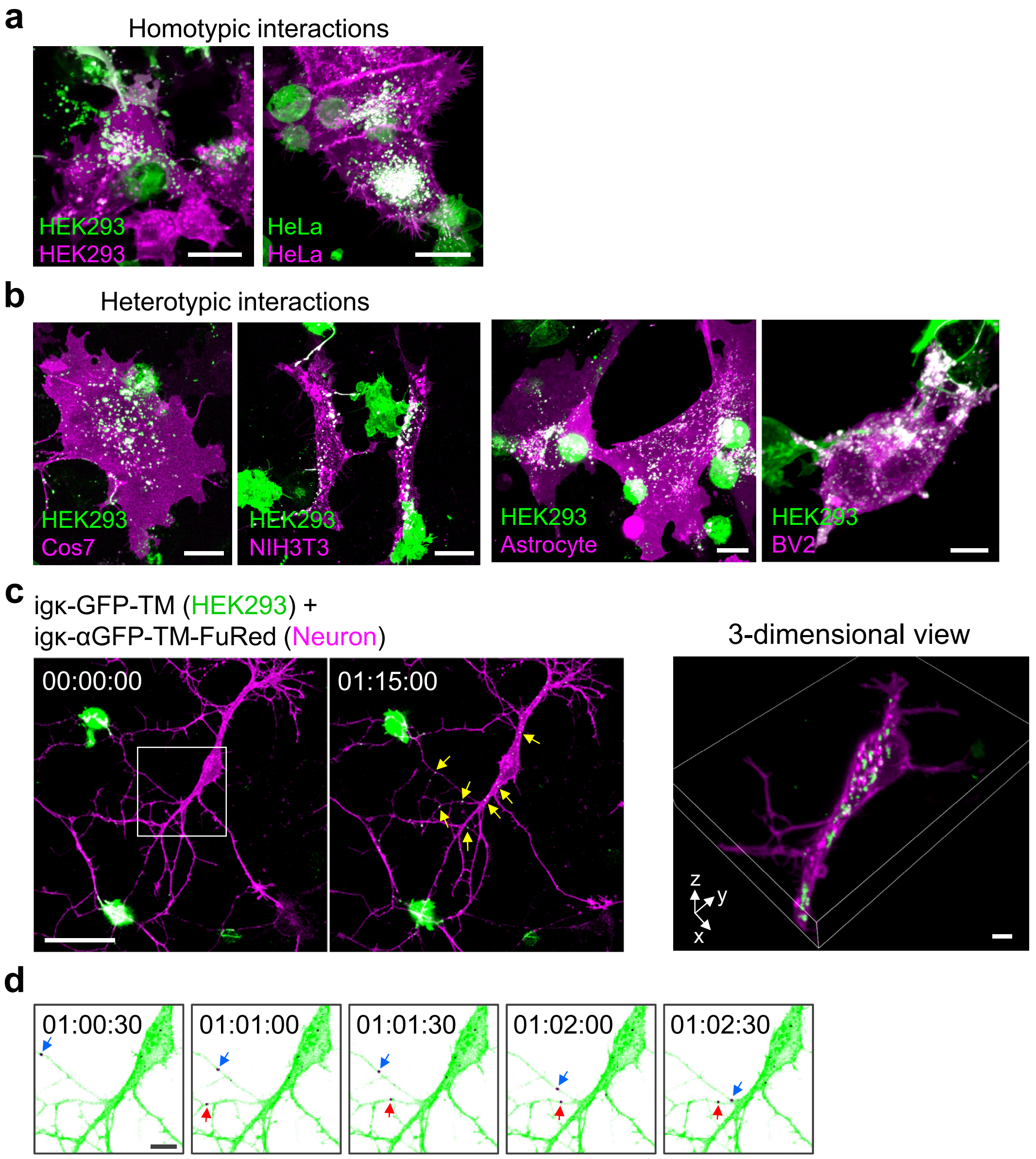

Supplementary Fig. 5. Induction of SynTrogo in various cell types.

a, Fluorescence images showing SynTrogo by homotypic cell-cell interactions. Scale bars, 20 µm. **b,** Fluorescence images showing SynTrogo by heterotypic cell-cell interactions. Scale bars, 20 µm. **c,** SynTrogo of HEK293T cells by cultured hippocampal neuron (left). A 3D-rendered image showing the accumulation of engulfed GFP molecules in neuronal soma and proximal neurites (right). Scale bars, 50 (left), 20 (right) µm. **d,** Time-lapse images showing trafficking of GFP-positive vesicles within neurites. Black dots (highlighted by blue and red arrows) indicate tracked vesicles. Time is indicated as hours:minutes:seconds. Scale bar, 10 µm.

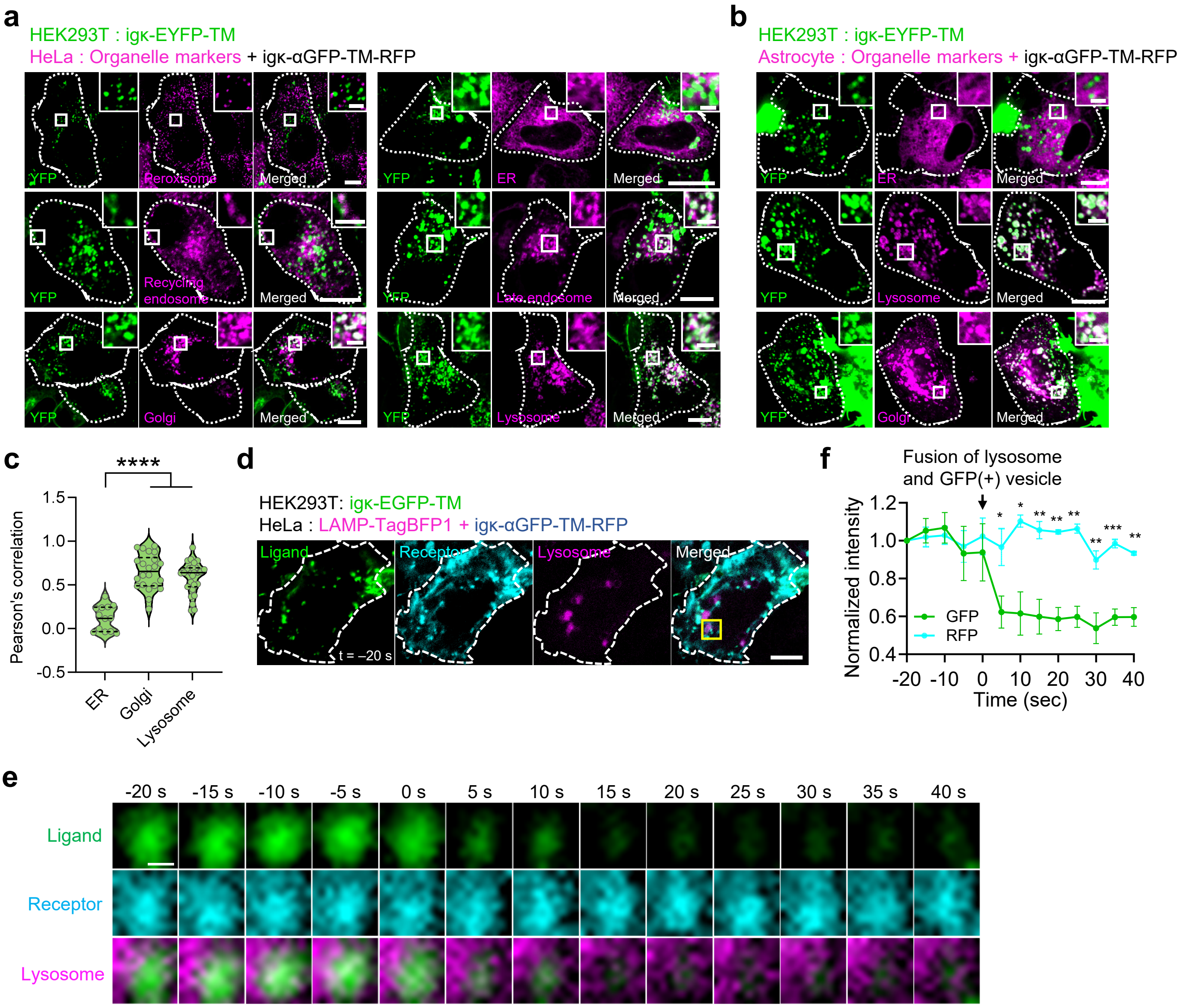

Supplementary Fig. 6. Subcellular localizations of engulfed ligand molecules upon SynTrogo.

a, Fluorescence Images showing subcellular localizations of engulfed ligands. HEK293T cells expressing EYFP-fused ligand were co-incubated with HeLa cells expressing the receptor and CFP-labeled organelle markers. Scale bars, 20 μm (large), 5 μm (inset). **b,** Fluorescence images of cultured astrocytes showing subcellular localizations of engulfed ligands. Scale bars, 20 μm. **c,** Pearson's correlation analysis showing colocalization of engulfed ligands and organelles markers in astrocytes. n = 13 (ER), 22 (Golgi), and 27 (Lysosome) cells. **d,** Fluorescence images showing subcellular localizations of lysosomes and engulfed ligand-vesicles in receptor-expressing HeLa cells. Scale bar, 10 μm. **e,** Time-lapse imaging of the fusion event between a vesicle containing both ligand and receptor and a lysosome, within the yellow boxed region in **d**. Time points are aligned relative to the moment of fusion (t = 0 s). Scale bar, 0.5 μm. **f,** Quantification of GFP and RFP fluorescence intensities during lysosomal fusion. n = 3 events. Data are presented as median with upper and lower quartiles (25th and 75th percentiles; dotted lines) in **c**, and as mean ± s.e.m. in **f**. Statistical significance was determined using One-way ANOVA followed by Tukey's multiple comparison test for **c** and two-way repeated-measures ANOVA followed by Tukey's multiple comparison test for **f**. *P < 0.05, **P < 0.01, ***P < 0.001, ****P < 0.0001.

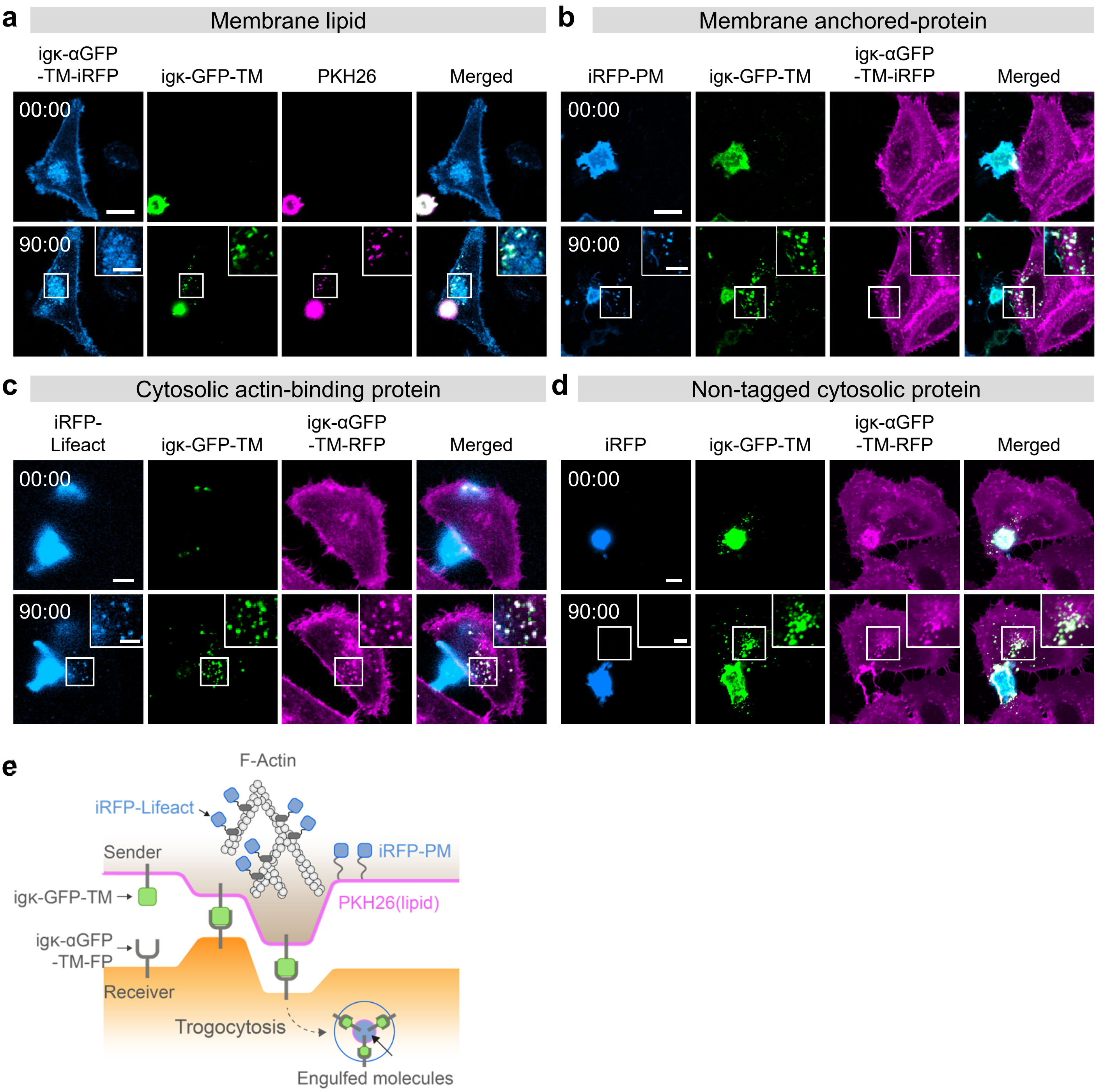

Supplementary Fig. 7. Co-transfer of various subcellular molecules with the ligand during SynTrogo. **a-d,** Fluorescence images showing the uptake of various molecules along with the ligand: **a,** Membrane lipid (labeled with 10 μM PKH26 dye), **b,** Membrane-anchored protein (iRFP-PM), **c,** Cytosolic actin-binding (iRFP-Lifeact) proteins. **d,** Non-tagged cytosolic fluorescent protein (iRFP) expressed in ligand-positive cells. Scale bars, 10 μm (large), 5 μm (inset) **e,** Schematic summarizing the results of co-transfer during SynTrogo. Scale bars 20 μm.

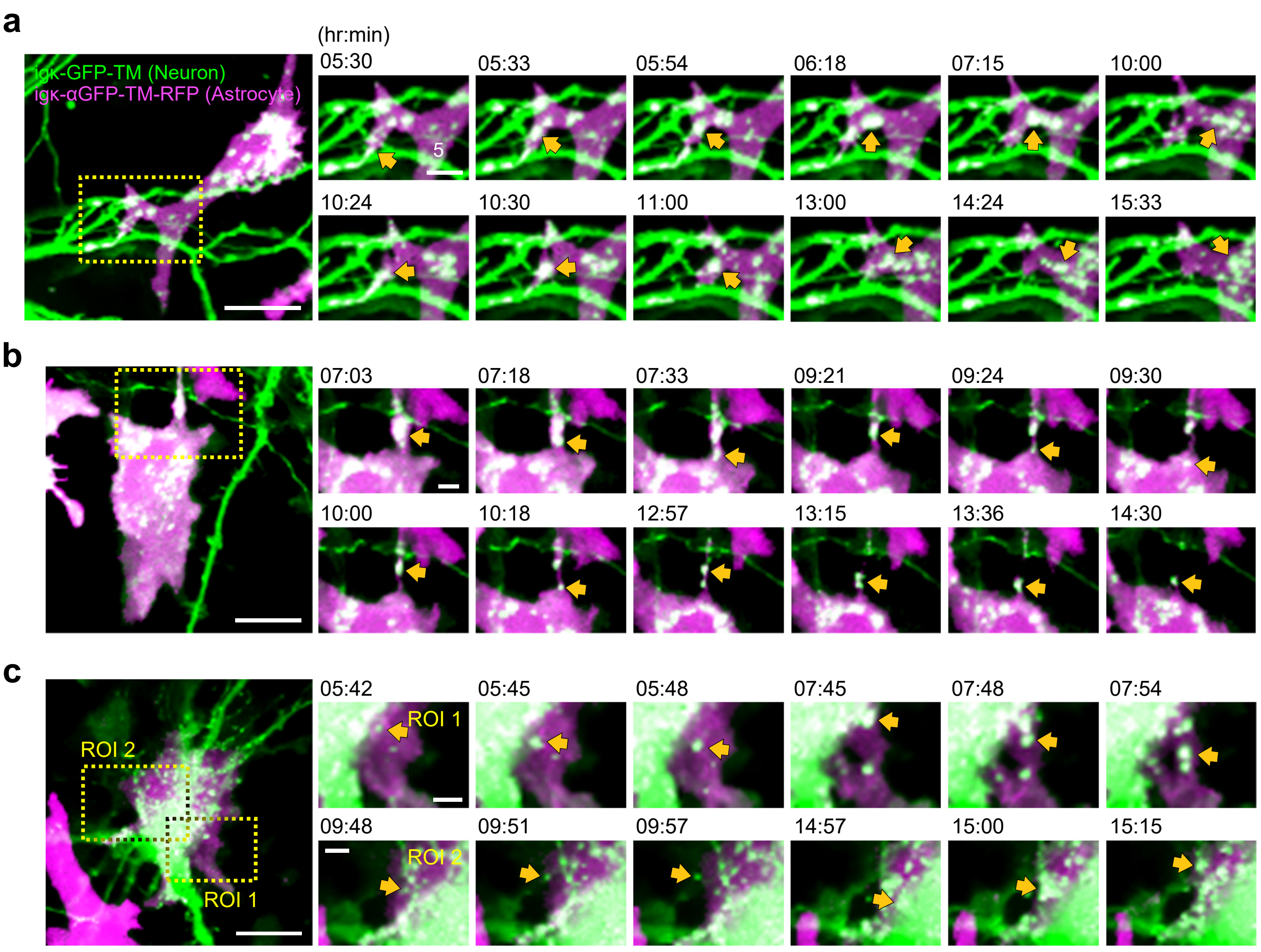

Supplementary Fig. 8 Real-time monitoring of uptake of ligand-labeled, distinct neuronal submembrane compartments by receptor-expressing astrocytes.

Cultured hippocampal neurons expressing the ligand and astrocytes expressing the receptor were co-incubated for 16 hours. Fluorescence images were captured every 3 min. **a-c,** SynTrogo of ligand-expressing neuronal membrane compartments by receptor-expressing astrocytes: **(a)** dendrites, **(b)** axon, and **(c)** soma. Scale bars, 20 µm (left), 5 µm (right, magnified images). Time is indicated as hours:minutes.

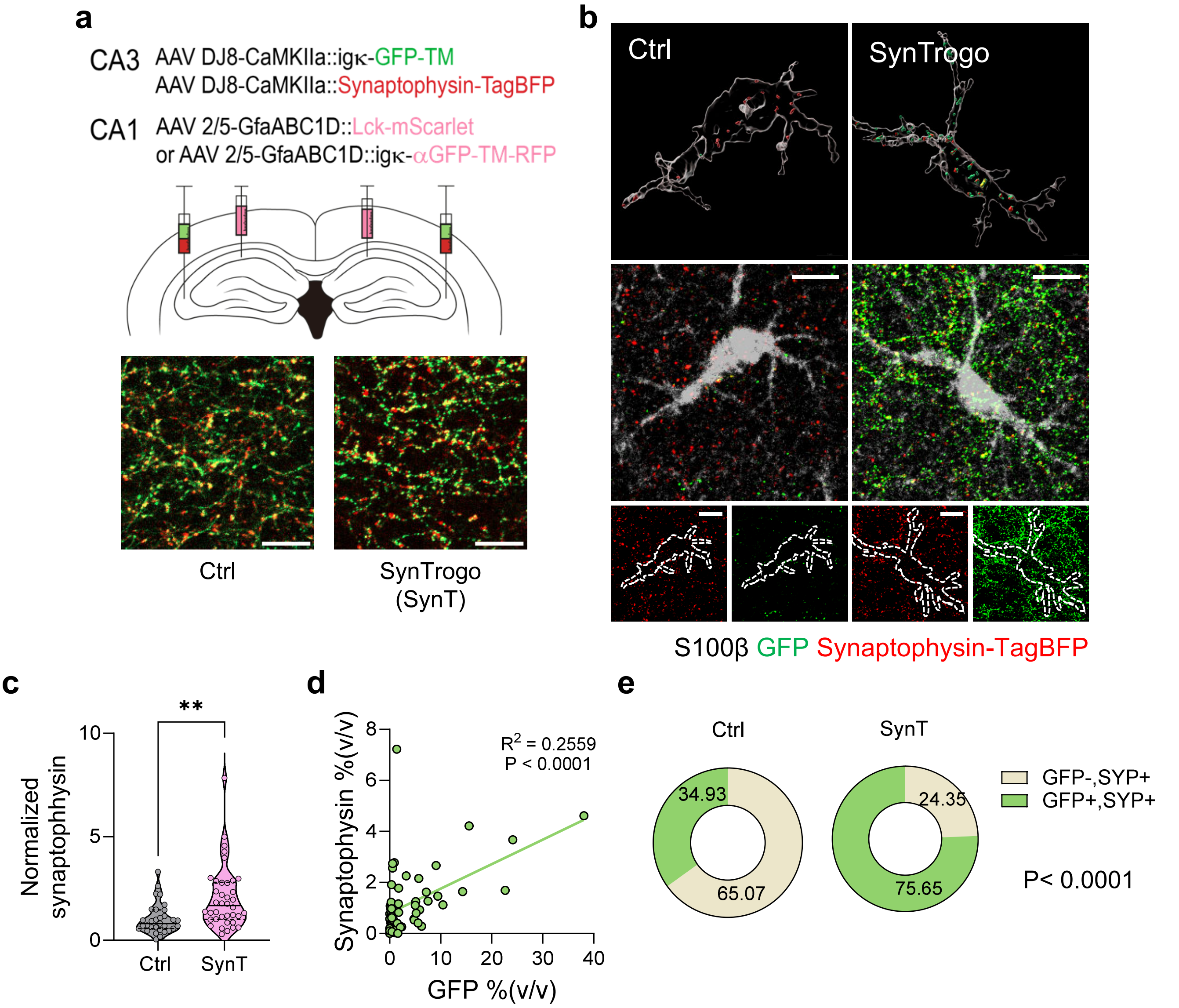

Supplementary Fig. 9 Localization of exogenously expressed synaptophysin within astrocyte territories under SynTrogo.

a, Schematic of virus injection (top). To assess the localization of synaptic molecules within astrocytic territories, viruses encoding Synaptophysin-TagBFP and the GFP ligand were co-injected into CA3. In CA1, either Lck-mScarlet (Control) or the receptor (SynTrogo) virus was injected into the left or right hemisphere, respectively. Fluorescence images showing ligand and receptor in CA1 under Control and SynTrogo conditions (bottom). Scale bar 10 µm. **b,** 3D-rendered images (top) and confocal images (bottom) showing GFP ligand (green) and Synaptophysin-TagBFP (red) signals within astrocytic soma territories. Dotted lines indicate astrocytic soma territories visualized by S100β staining. Note that some GFP puncta visible in the confocal images were not retained in the 3D-rendered images, likely because puncta positioned above or below the astrocytic surface were removed during masking, or signals below the fluorescence intensity threshold were excluded during 3D reconstruction. Scale bars, 10 µm. **c,** Quantification of Synaptophysin-TagBFP signals within astrocytic soma territories. N = 3 (ctrl), 3 (SynT) mice; n = 20 (Ctrl), 26 (SynT) astrocytes. **d,** Correlation between GFP puncta and Synaptophysin-TagBFP in SynTrogo astrocytes. Linear regression analysis performed on n = 60 astrocytes; coefficient of determination (R^2) = 0.2559, P < 0.0001. **e,** Pie charts showing the proportion of Synaptophysin (SYP) signals with (green) or without (yellow) GFP located within astrocytic soma territories. Data are presented Data are presented as median with upper and lower quartiles (25th and 75th percentiles; dotted lines) in **c**. Statistical significance was determined by unpaired *t*-test (**c**) and chi-square test (**e**). *$P < 0.05$, ****$P < 0.0001$.

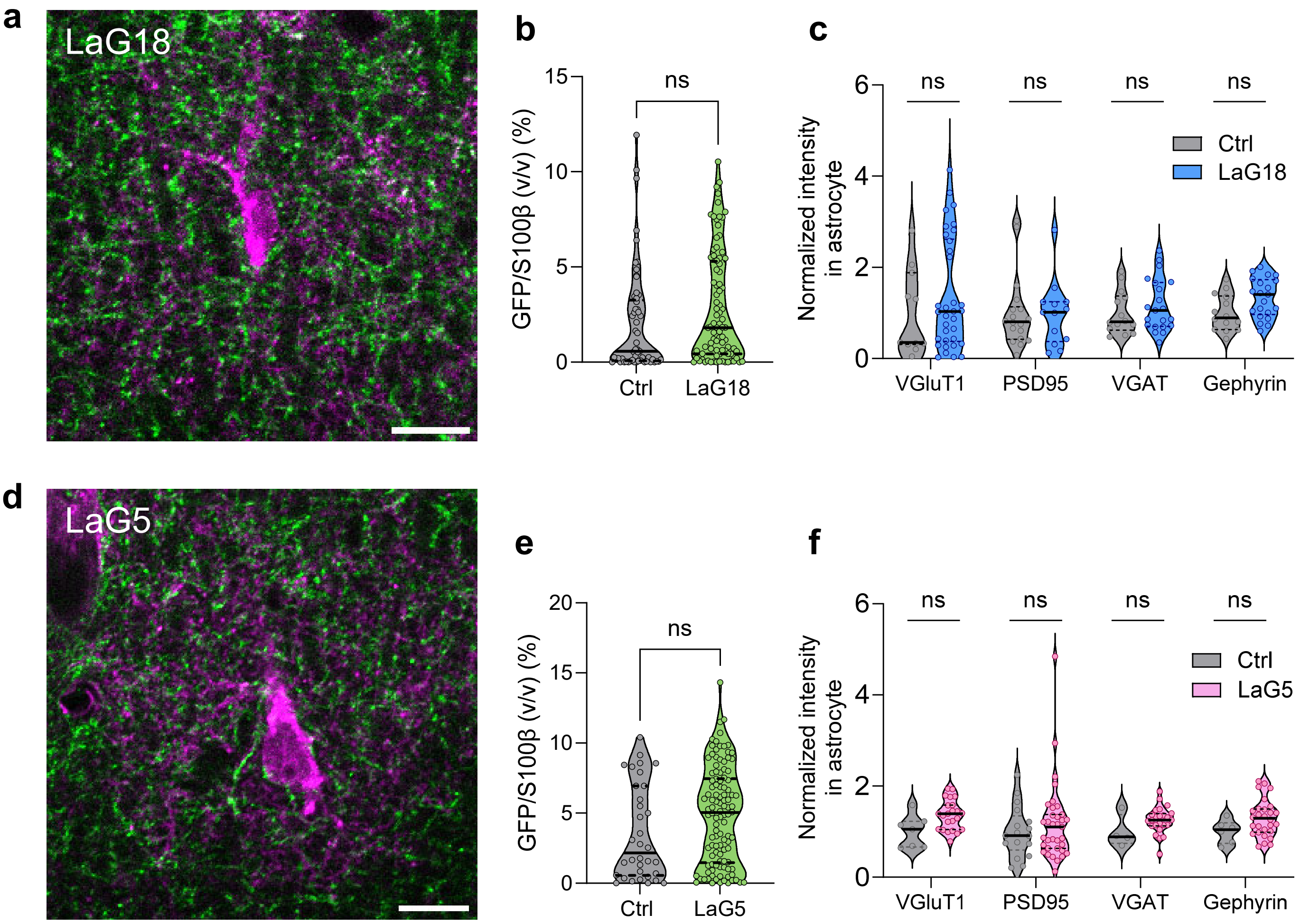

Supplementary Fig. 10. Low-affinity GFP receptors failed to accumulate endogenous synaptic molecules within astrocytic territories.

a, Representative images showing axons expressing the GFP ligand and astrocytes expressing the LaG18-receptor. **b,** Quantification of the volume ratio (v/v) of GFP ligand localized within astrocytic soma territories relative to astrocytic soma (S100β-positive). **c,** Quantification of normalized fluorescence intensities of synaptic molecules localized within astrocytic soma territories. N = 2 (Ctrl), 2 (LaG18) mice; n = 57 (Ctrl), 88 (LaG18) total cells; control group: n = 11 (VGluT1), 18 (PSD95), 14 (VGAT), 14 (Gephyrin) cells; LaG18 group: n = 34 (VGluT1), 13 (PSD95), 21 (VGAT), 21 (Gephyrin) cells. **d,** Representative images showing axons expressing the GFP ligand and astrocytes expressing the LaG5-receptor. **e,** Quantification of the volume ratio (v/v) of GFP ligand localized within astrocytic soma territories relative to astrocytic soma (S100β-positive). **f,** Quantification of normalized fluorescence intensities of synaptic molecules localized within astrocytic soma territories. N = 2 (Ctrl), 2 (LaG5) mice; n = 34 (Ctrl), 110 (LaG5) total cells; control group: n = 7 (VGluT1), 17 (PSD95), 4 (VGAT), 6 (Gephyrin) cells; LaG5 group: n = 23 (VGluT1), 35 (PSD95), 24 (VGAT), 28 (Gephyrin) cells. Scale bars, 10 μm. Data are presented as median with upper and lower quartiles (25th and 75th percentiles; dotted lines) in **b**, **c**, **e**, and **f**. Statistical significance was determined using unpaired t-test for **b,e** and two-way ANOVA followed by Uncorrected Fisher's LSD test for **c,f**. ns, not significant.

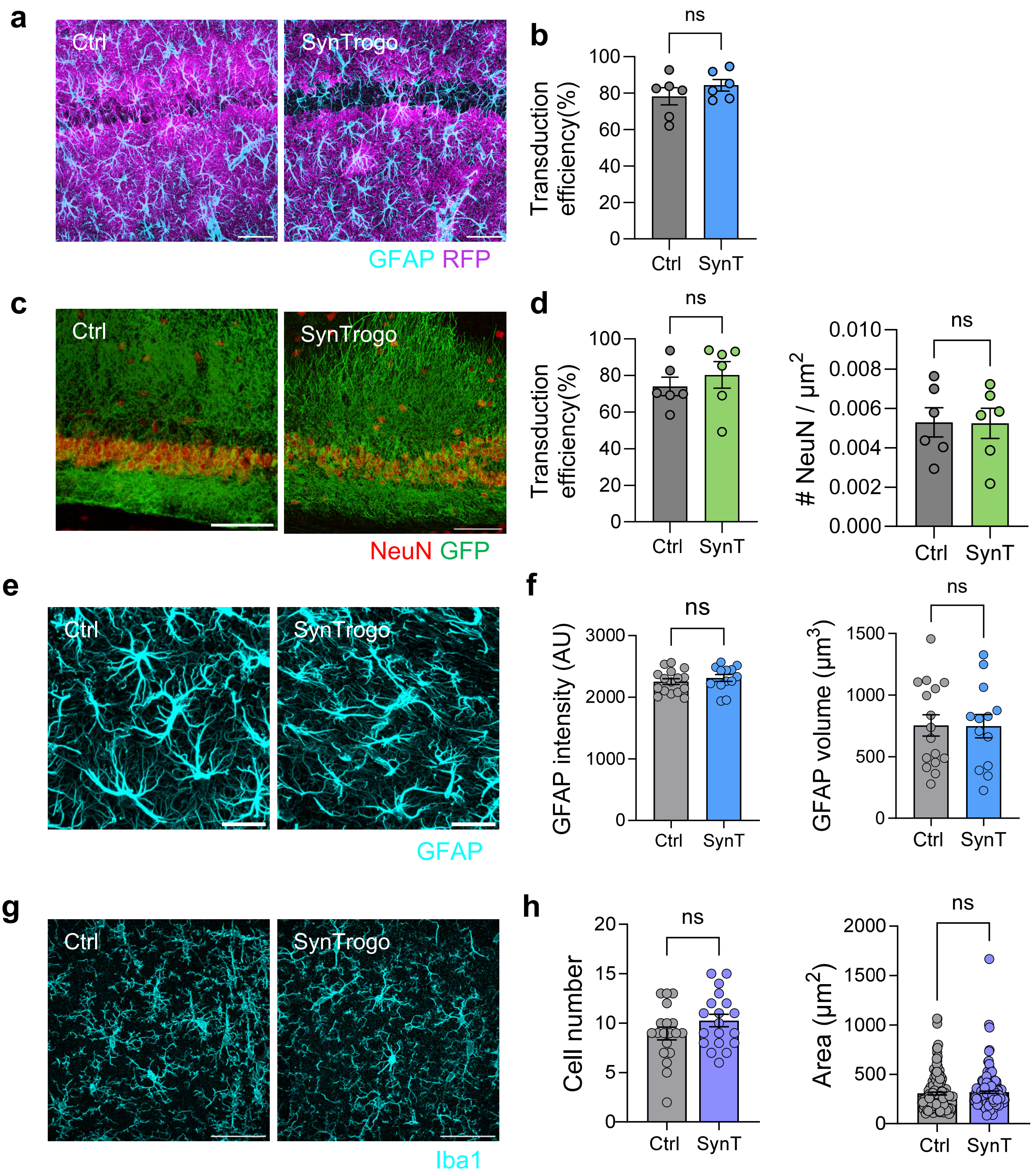

Supplementary Fig. 11. Assessment of viral transduction efficiency and lack of glial reactivity changes under SynTrogo (SynT).

a, Representative images showing viral expression in CA1 astrocytes. GFAP signals (cyan) overlap with astrocytes. Scale bar, 50 μm. **b**, Quantification of viral transduction efficiency in astrocytes. N = 6 (Ctrl), 6 (SynT) mice. **c**, Representative images showing viral expression in CA3 neurons. NeuN signals (red) overlap with neurons. Scale bar, 100 μm. **d**, Quantification of viral transduction efficiency in neurons (left) and neuronal density (NeuN-positive cells per μm^2) (right). N = 6 (Ctrl), 6 (SynT) mice. **e**, Representative fluorescence images of GFAP immunostaining in the CA1 region. Scale bar, 20 μm. **f,** Quantification of the intensity (left) and volume (right) of GFAP signals. N = 3 (Ctrl), 3 (SynT) mice; n = 16 (Ctrl), 13 (SynT) cells. **g,** Fluorescence images showing Iba1 signals in the CA1 region. Scale bar, 50 μm. **h,** Quantification of the number (left) and area (right) of microglia. N = 3 (Ctrl), 3 (SynT) mice; (left) n = 19 imaging areas per group; (right) n = 165 (Ctrl), 188 (SynT) cells. Data are presented as mean ± s.e.m. Statistical significance was determined using unpaired *t*-test. ns, not significant.

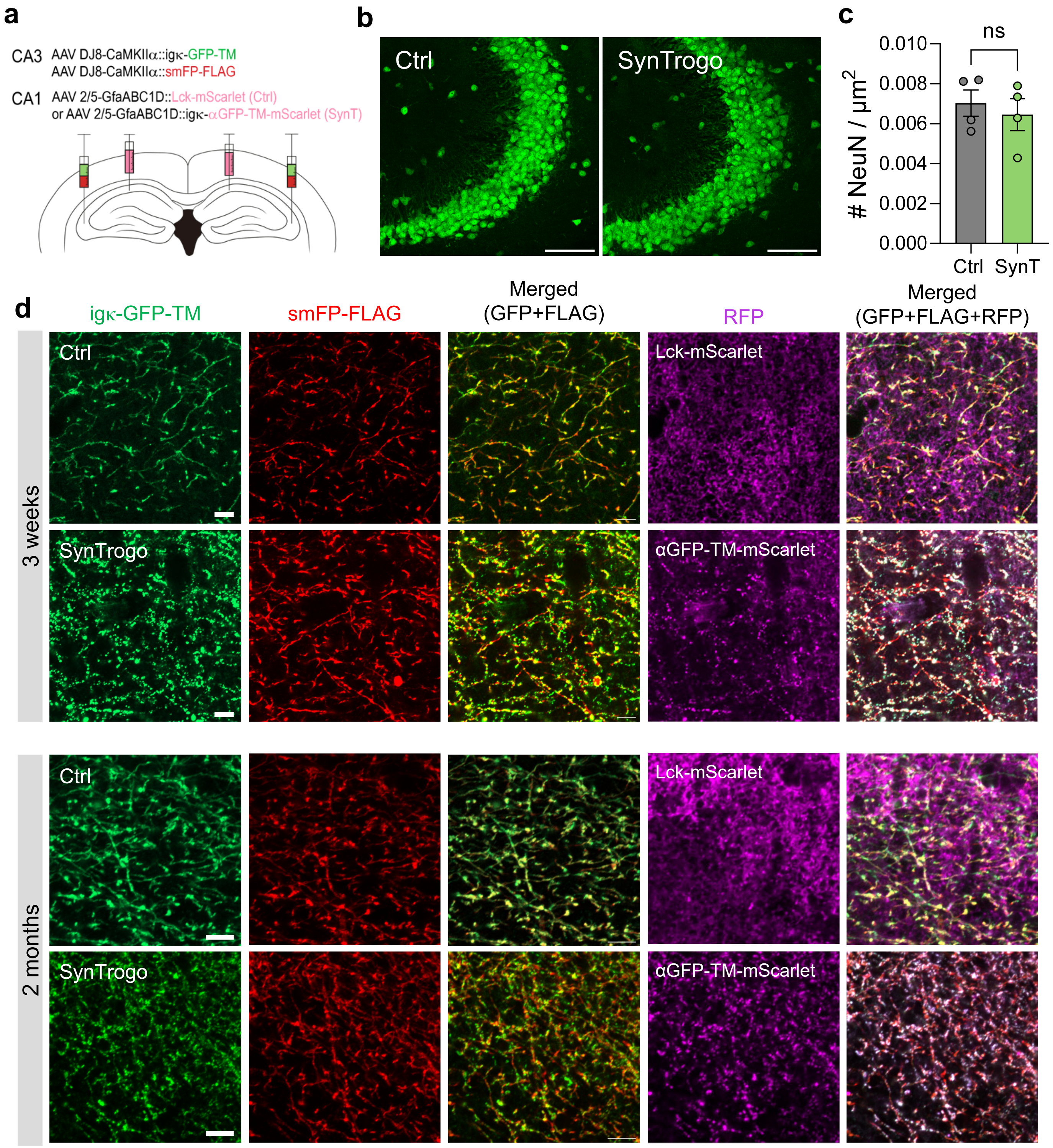

Supplementary Fig. 12. SynTrogo did not induce neuronal or axonal loss at 3 weeks and 2 months.

a, Schematic illustrating viral injections used to visualize axon morphology. Two AAVs were co-injected into the CA3 region: AAV-CaMKIIα-igκ-GFP-TM (ligand) and AAV-CaMKIIα-smFP-FLAG (cell filling marker). In CA1, either AAV-GfaABC1D-Lck-mScarlet (control) or AAV-GfaABC1D-igκ-αGFP-TM-mScarlet (SynTrogo) was injected. Axon morphology and NeuN density were analyzed at 3 weeks and 2 months post-injection. **b,** Representative images of NeuN immunostaining in the CA1 region 2 months after SynTrogo (SynT) viral injection. Scale bar, 100 μm. **c**, Quantification of neuronal density (NeuN-positive cells per μm^2). N = 4 (Ctrl), 4 (SynT) mice. **d**, Representative images of Schaffer collateral axons visualized by expression of membrane-anchored ligand (igk-GFP-TM) and cytosolic protein (smFP-FLAG) at 3 weeks (top) and 2 months (bottom). Scale bars, 5 μm. Data are presented as mean ± s.e.m. Statistical significance was determined using unpaired *t*-test. ns, not significant.

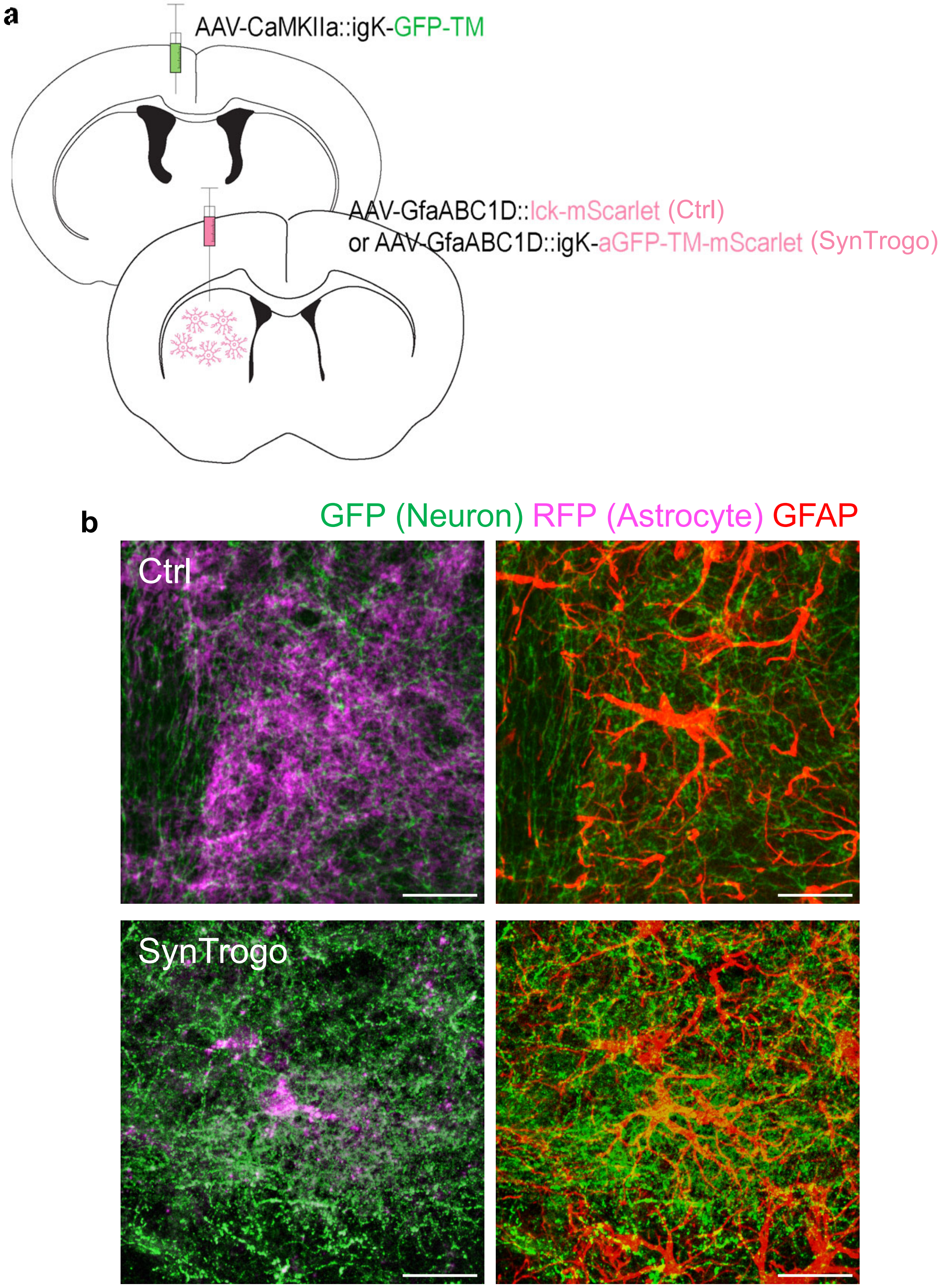

Supplementary Fig. 13. Application of SynTrogo in the motor cortex–striatum circuit.

a, Schematic diagram showing targeting of the motor cortex-striatum circuit. AAV-CaMKIIα-igκ-GFP-TM (ligand) was injected into motor cortex neurons projecting to the striatum. Striatal astrocytes were transduced with either AAV-GfaABC1D-Lck-mScarlet (control) or AAV-GfaABC1D-igκ-αGFP-TM-mScarlet (SynTrogo). **b**, Representative images of striatal astrocytes in control (top) and SynTrogo (bottom) conditions. Scale bar, 20 µm.

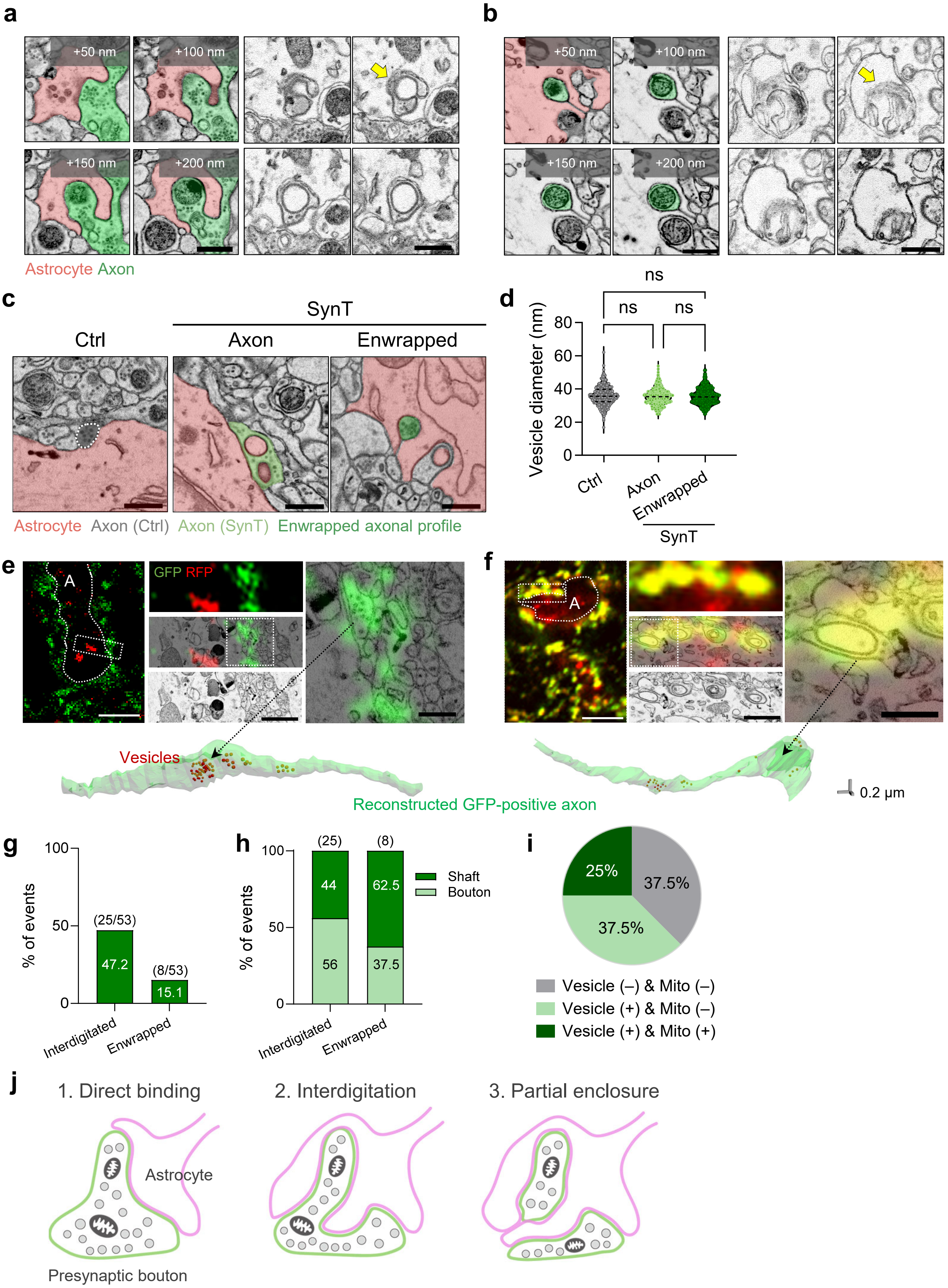
a
+50 nm
+100 nm
+150 nm
+200 nm
Astrocyte Axon
b
+50 nm
+100 nm
+150 nm
+200 nm
c
SynT
Ctrl
Axon
Enwrapped
Astrocyte Axon (Ctrl) Axon (SynT) Enwrapped axonal profile
d
ns
ns
ns
Vesicle diameter (nm)
80
60
40
20
0
Ctrl
Axon
Enwrapped
SynT
e
A
GFP RFP
f
A
Vesicles
Reconstructed GFP-positive axon
0.2 μm
g
% of events
100
50
0
(25/53)
47.2
(8/53)
15.1
Interdigitated
Enwrapped
h
% of events
100
50
0
(25)
(8)
44
62.5
56
37.5
Shaft
Bouton
Interdigitated
Enwrapped
i
25%
37.5%
37.5%
Vesicle (–) & Mito (–)
Vesicle (+) & Mito (–)
Vesicle (+) & Mito (+)
j
1. Direct binding
2. Interdigitation
3. Partial enclosure
Astrocyte
Presynaptic bouton

Supplementary Fig. 14. Ultrastructural features at neuron-astrocyte interfaces under SynTrogo.

a, Sequential EM images showing astrocytic processes forming interdigitated configurations at presynaptic bouton–astrocyte interfaces under SynTrogo (left), and the occasional co-occurrence of a phagolysosome-like structure (arrow, right). Scale bars, 0.5 µm. **b**, Sequential EM images depicting membrane-enwrapped axonal profiles observed in single 2D sections, in which astrocytic processes partially surround presynaptic material and display narrowed intermembrane spacing under SynTrogo (left). Phagolysosome-like structures (indicated by arrow) were occasionally observed near these membrane configurations (right). Scale bars, 0.5 µm. **c**, Representative EM images showing presynaptic vesicles within axons and membrane-enwrapped axonal profiles located within astrocytic territories. **d,** Quantification of vesicle diameters in axons and in membrane-enwrapped axonal profiles identified in 2D EM sections. N = 3 (Ctrl), 3 (SynT) mice; n = 235 (Ctrl axonal vesicles), 283 (SynT axonal vesicles), 204 (enwrapped profile vesicles). **e-f,** Correlative light and electron microscopy (CLEM) images showing 3D-reconstructed GFP-positive axons adjacent to astrocytes in control **(e)** and SynT **(f)** conditions, confirming continuity of enwrapped profiles with parent axons. Scale bars, 5 µm (left), 1 µm (middle), 0.5 µm (right). **g,** Proportion of axons exhibiting interdigitated or membrane-enwrapped configurations among 53 axons analyzed under SynTrogo conditions. **h,** Proportion of boutons and shafts containing interdigitated or membrane-enwrapped configurations. **i,** Proportion of membrane-enwrapped profiles containing vesicles and/or mitochondria. N = 3 mice; n = 3 astrocytes, 8 enwrapped profiles. **j**, Schematic illustration of SynTrogo-associated membrane interactions and related structural features at neuron-astrocyte interfaces. Data are presented as median with upper and lower quartiles (25th and 75th percentiles; dotted lines) in **d**. Statistical significance was determined using one-way ANOVA followed by Tukey's multiple comparison test for **d**. ns, not significant.

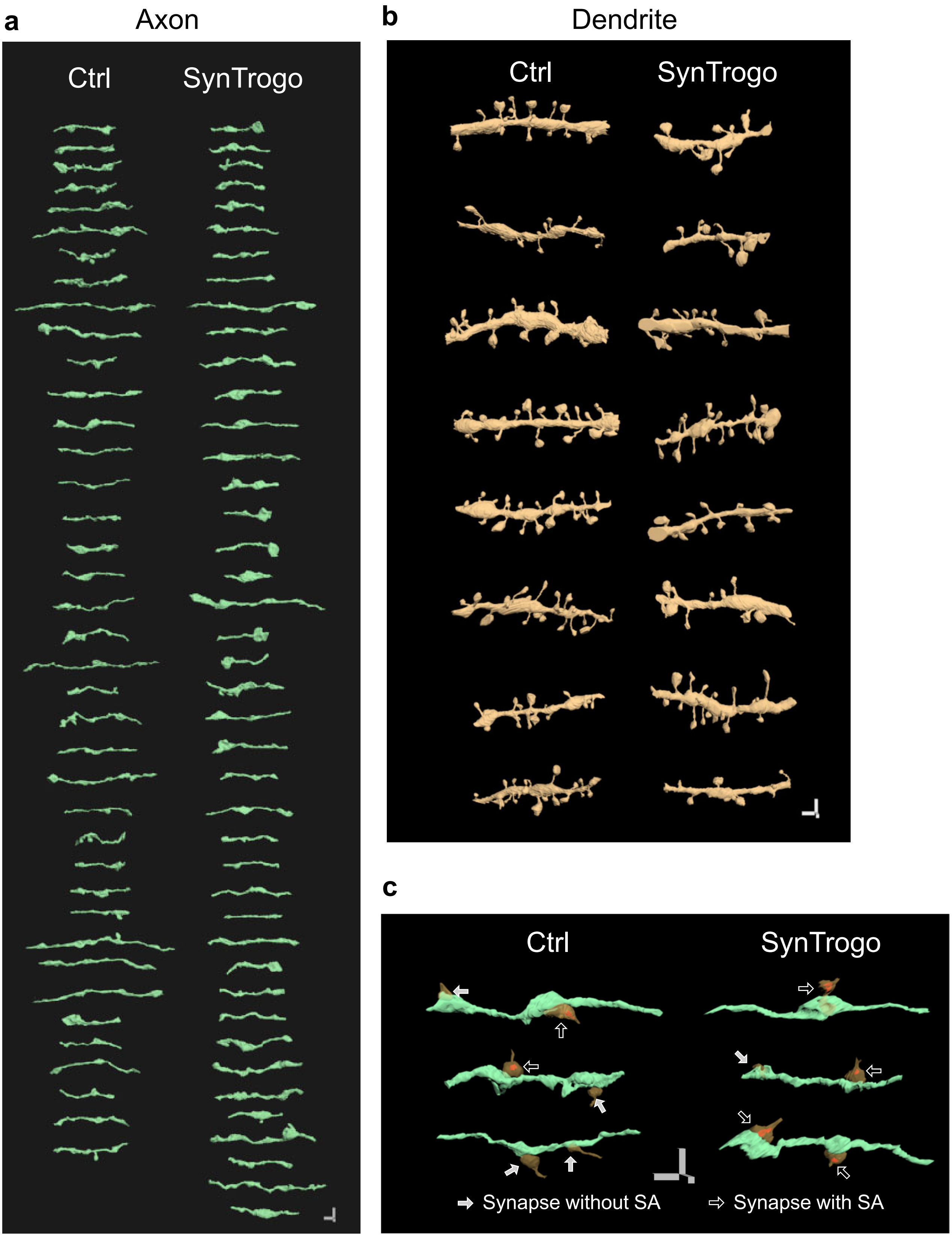

Supplementary Fig. 15. Ultrastructural analysis of axons, dendrites, and spines.

a-b, 3D-reconstructed EM images showing the structure of **(a)** ligand-labeled axons (n = 39 (Ctrl), 42 (SynTrogo) axons), and **(b)** coupled dendrites (n = 8 dendrites for each condition). **c,** 3D-reconstructed EM images showing presynaptic boutons (green) and innervated spines (brown), and the location of spine apparatus (SA) (red). Scale bars, 1 µm.

a

Group	GFP ligand	Axonal GFP expression	Receptor-expressing astrocytes	Astrocyte contact (≤ 100 nm)
Ctrl GFP(+) axons	Present	GFP-positive	Absent	Yes
SynT GFP(+) axons	Present	GFP-positive	Present	Yes
SynT GFP(-) axons	Absent	GFP-negative	Present	Yes

b

Control axons	Axon length	Synapse #	Synapse # / µm
axon 01	5.72	2	0.350
axon 02	5.26	2	0.380
axon 03	6.49	4	0.616
axon 04	5.84	2	0.343
axon 05	7.65	2	0.261
axon 06	10.85	3	0.277
axon 07	5.98	2	0.335
axon 08	7.07	3	0.425
axon 09	12.64	3	0.237
axon 10	9.42	2	0.212
axon 11	4.79	2	0.418
axon 12	7.21	1	0.139
axon 13	8.19	2	0.244
axon 14	7.09	2	0.282
axon 15	6.74	1	0.148
axon 16	6.83	2	0.293
axon 17	5.62	2	0.356
axon 18	4.80	3	0.625
axon 19	5.88	2	0.340
axon 20	7.91	3	0.379
axon 21	6.42	2	0.312
axon 22	13.29	2	0.150
axon 23	4.90	1	0.204
axon 24	8.60	2	0.233
axon 25	7.45	2	0.269
axon 26	9.94	3	0.302
axon 27	6.04	2	0.331
axon 28	27.79	9	0.324
axon 29	5.79	2	0.345
axon 30	4.93	2	0.406
axon 31	5.50	2	0.363
axon 32	5.36	1	0.187
axon 33	14.04	4	0.285
axon 34	10.63	3	0.282
axon 35	24.83	6	0.242
axon 36	5.28	2	0.379
axon 37	5.98	2	0.334
axon 38	8.61	3	0.348
axon 39	5.21	3	0.576
axon 40	6.86	3	0.437
axon 41	7.16	3	0.419
axon 42	5.51	2	0.363
axon 43	4.74	2	0.422
axon 44	5.74	2	0.348
axon 45	5.49	3	0.546
axon 46	7.34	4	0.545
axon 47	8.84	4	0.452
axon 48	4.04	1	0.248
axon 49	5.21	3	0.576
Average	7.87	2.56	0.35

c

SynTrogo GFP(+) axons	Axon length	Synapse #	Synapse # / µm
axon 01	4.72	3	0.636
axon 02	5.80	1	0.172
axon 03	4.82	2	0.415
axon 04	4.21	1	0.238
axon 05	4.57	1	0.219
axon 06	4.56	1	0.219
axon 07	6.52	2	0.307
axon 08	5.26	2	0.380
axon 09	11.26	4	0.355
axon 10	6.10	1	0.164
axon 11	5.45	2	0.367
axon 12	7.47	2	0.268
axon 13	4.71	1	0.212
axon 14	8.47	2	0.236
axon 15	6.31	1	0.158
axon 16	8.72	1	0.115
axon 17	5.21	2	0.384
axon 18	21.85	4	0.183
axon 19	4.38	1	0.228
axon 20	5.81	1	0.172
axon 21	4.22	2	0.474
axon 22	12.59	3	0.238
axon 23	4.92	1	0.203
axon 24	4.60	1	0.218
axon 25	7.53	2	0.266
axon 26	6.94	1	0.144
axon 27	7.24	3	0.414
axon 28	5.29	0	0.000
axon 29	8.33	1	0.120
axon 30	5.16	0	0.000
axon 31	5.35	0	0.000
axon 32	7.44	0	0.000
axon 33	5.30	0	0.000
axon 34	8.26	3	0.363
axon 35	5.06	1	0.198
axon 36	6.03	0	0.000
axon 37	6.06	0	0.000
axon 38	5.64	1	0.177
axon 39	7.06	2	0.283
axon 40	8.19	1	0.122
axon 41	7.55	2	0.265
axon 42	5.25	1	0.190
axon 43	9.93	2	0.201
axon 44	5.99	1	0.167
axon 45	11.76	1	0.085
axon 46	6.29	1	0.159
axon 47	6.82	1	0.147
axon 48	7.54	2	0.265
axon 49	8.31	1	0.120
axon 50	6.96	2	0.287
axon 51	7.52	1	0.133
axon 52	6.34	1	0.158
axon 53	7.18	1	0.139
Average	6.88	1.40	0.21

d

SynTrogo GFP(-) axons	Axon length	Synapse #	Synapse # / µm
axon 01	4.72	3	0.636
axon 02	5.80	1	0.172
axon 03	4.82	2	0.415
axon 04	4.21	1	0.238
axon 05	4.57	1	0.219
axon 06	4.56	1	0.219
axon 07	6.52	2	0.307
axon 08	5.26	2	0.380
axon 09	11.26	4	0.355
axon 10	6.10	1	0.164
axon 11	5.45	2	0.367
axon 12	7.47	2	0.268
axon 13	4.71	1	0.212
Average	6.78	2.58	0.39

Supplementary Fig. 16. Summary of features and selection criteria for all analyzed axons.

a, Definition of experimental groups used for CLEM analysis. Control GFP(+) axons were GFP-positive axons in animals without receptor expression in astrocytes. SynTrogo GFP(+) axons were GFP-positive axons in proximity to receptor-expressing astrocytes. SynTrogo GFP(–) axons were GFP-negative axons located near receptor-expressing astrocytes. All analyzed axons were confirmed to be in close contact with astrocytic membranes (≤ 100 nm). **b–d,** Tables listing every axon analyzed in each group: **(b)** Control GFP(+) axons, **(c)** SynTrogo GFP(+) axons, and **(d)** SynTrogo GFP(–) axons. For each axon, the segment length (µm), number of annotated synapses, and calculated synaptic density (synapse number/µm) are listed. Axons were selected based on consistent proximity to astrocytes to minimize sampling bias, thereby ensuring consistent criteria across groups. Group averages are shown at the bottom of each panel.

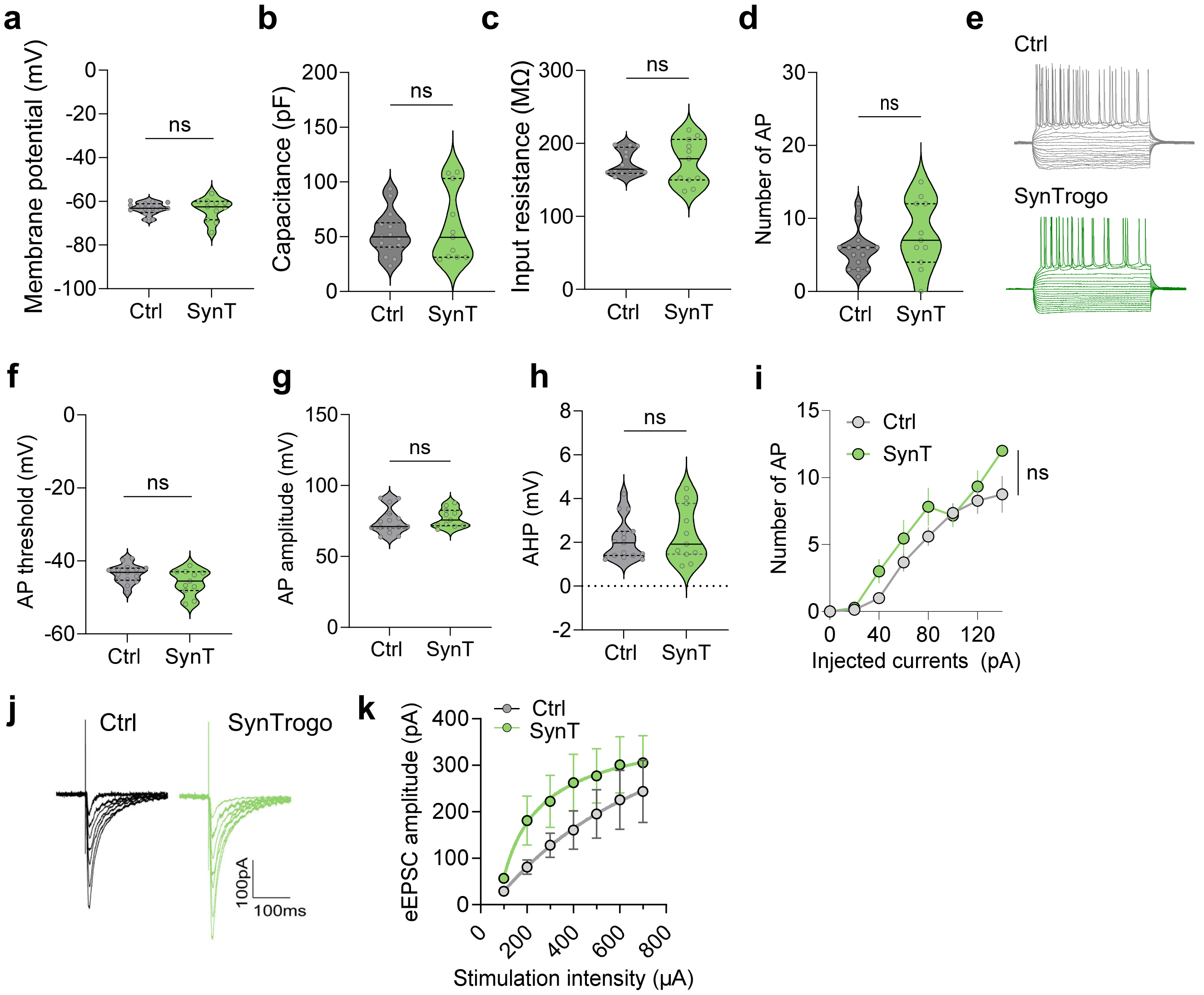

Supplementary Fig. 17. Electrophysiological analysis of CA1 pyramidal neurons under SynTrogo.

a-i, Intrinsic properties of CA1 pyramidal neurons under each condition (Ctrl and SynT) were measured. **(a)** Membrane potential. **(b)** Capacitance. **(d)** Input resistance. **(d)** Rheobase. **(e)** Representative action potential (AP) trances. **(f)** AP threshold. **(g)** AP amplitude. **(h)** AHP (after hyperpolarization) amplitude. **i,** Number of APs in response to injected currents. N = 5 (Ctrl), 4 (SynT) mice; n = 15 (Ctrl), 11 (SynT) neurons. **j,** Representative trace of evoked EPSC (eEPSC). **k,** Input-output curve of CA1 neurons. N = 5 (Ctrl), 5 (SynT) mice; n = 14 (Ctrl), 13 (SynT) neurons. Data are presented as median with upper and lower quartiles (25th and 75th percentiles; dotted lines) in **a-d**, **f-h**, and as mean ± s.e.m. in **i**, **k**. Statistical significance was determined using unpaired t-test for **a-d, f-h** and two-way ANOVA followed by Sidak's comparison test for **i, k.** ns, not significant.

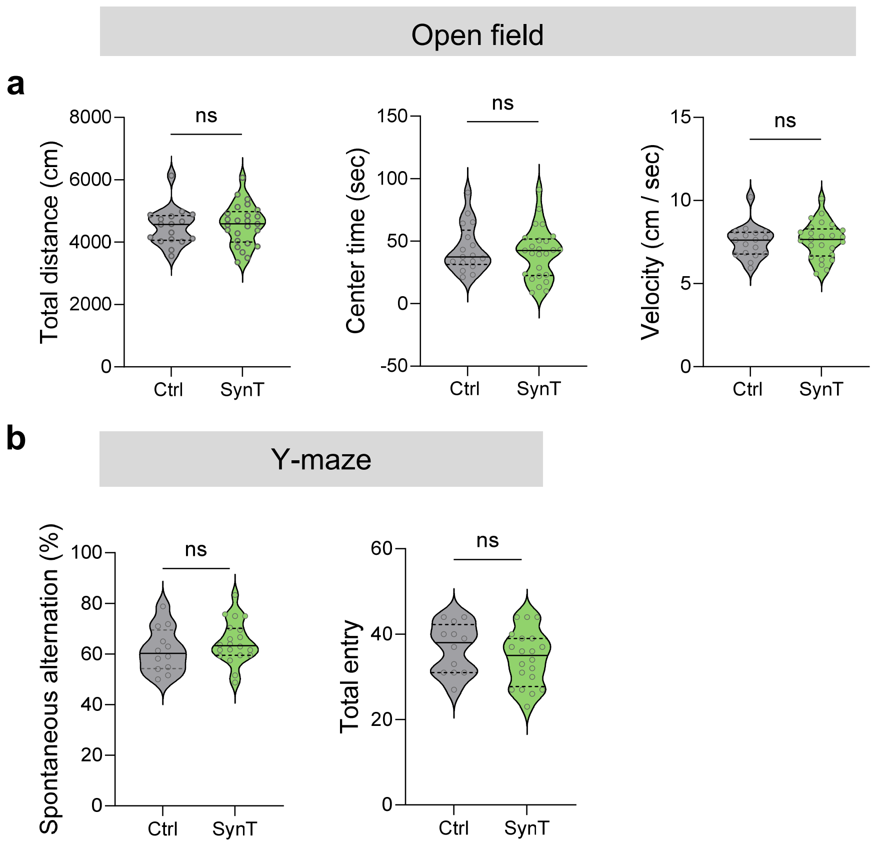

Supplementary Fig. 18. Assessment of locomotion, anxiety, and working memory following SynTrogo targeting of the CA3–CA1 pathway.

a, Open field test to measure total distance (left), velocity (middle), and duration (right) of locomotion. N = 17 (Ctrl), 25 (SynT) mice. **b,** Y-maze test to measure spontaneous alternation (left) and the number of total entry (right). N = 12 (Ctrl), 20 (SynT) mice. Data are presented as median with upper and lower quartiles (25th and 75th percentiles; dotted lines). Statistical significance was determined using unpaired *t*-test. ns, not significant.

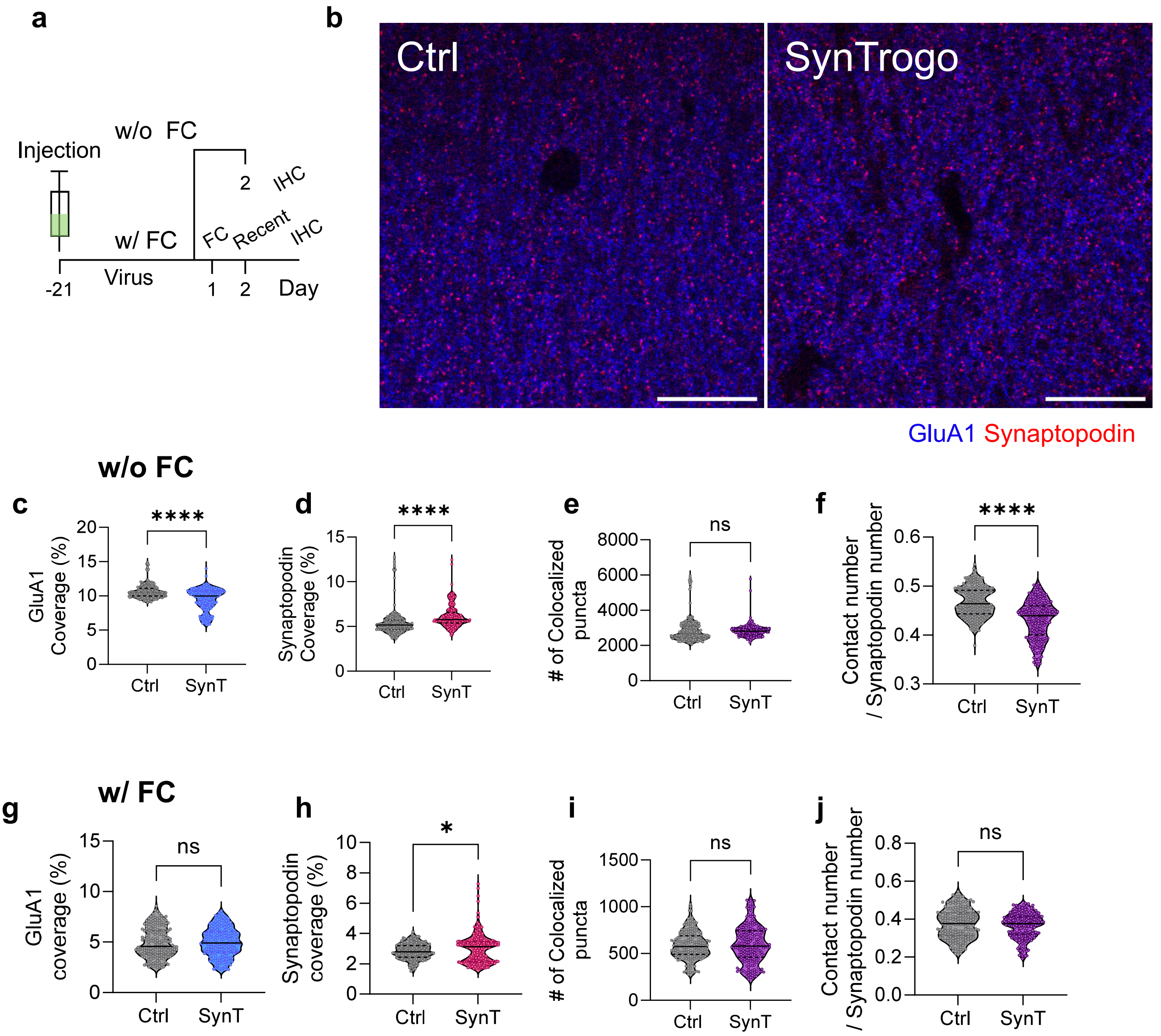

Supplementary Fig. 19. Relationship between GluA1 and synaptopodin signals in the presence and absence of fear conditioning.

a, Timeline illustrating the experimental schedule for immunohistochemistry (IHC) experiments. **b**, Representative confocal images showing GluA1 (blue) and synaptopodin (red) immunostaining with merged images. Scale bar, 5 μm. **c-f**, Quantification of GluA1 and synaptopodin without fear conditioning in control and SynTrogo (SynT) groups: (**c**) GluA1 coverage, (**d**) synaptopodin coverage, (**e**) number of GluA1 and synaptopodin colocalized puncta, and (**f**) proportion of colocalized puncta among total synaptopodin puncta. N = 3 (Ctrl), 3 (SynT) mice. n = 242 (Ctrl), 242 (SynT) imaging areas. **g-j**, Quantification of GluA1 and synaptopodin after fear conditioning in control and SynT groups: (**g**) GluA1 coverage, (**h**) synaptopodin coverage, (**i**) number of GluA1 and synaptopodin colocalized puncta, and (**j**) proportion of colocalized puncta among total synaptopodin puncta. N = 6 (Ctrl), 6 (SynT) mice per group. n = 181 (Ctrl), 220 (SynT) imaging areas. Data are presented as median with upper and lower quartiles (25th and 75th percentiles; dotted lines). Statistical significance was determined using unpaired t-test. *$P < 0.05$, ****$P < 0.0001$; ns, not significant.
