# Supplementary material for: Remodeling synaptic connections via engineered neuron-astrocyte interactions
Source: Nat Commun. 2026 Apr 15;17:3490. doi: 10.1038/s41467-026-71440-w (PMC13084054; doi:10.1038/s41467-026-71440-w)
Supplement: Supplementary file 2 — Description of Additional Supplementary Files [file 41467_2026_71440_MOESM2_ESM.pdf]

Description of Additional Supplementary Files

Supplementary Movie 1. Time-lapse imaging of SynTrogo induced by ligand–receptor interaction. Ligand-expressing HEK293T cells were coincubated with receptorexpressing HeLa cells for 5 h. Fluorescent images were captured every 3 min. Numbers indicate hours:minutes:seconds. Scale bar, 100 μm.

Supplementary Movie 2. High-magnification timelapse imaging of SynTrogo. Magnified view of ligandexpressing HEK293T cells co-incubated with receptorexpressing HeLa cells. Fluorescent images were captured every 5 s. Numbers indicate minutes:seconds. Scale bar, 10 μm.

Supplementary Movie 3. Volumetric time-lapse imaging of SynTrogo. Ligand-expressing HEK293T cells were coincubated with receptorexpressing HeLa cells. Z- stack images were acquired every 3 min and rendered in 3D using the surface function in Imaris. Numbers indicate hours:minutes:seconds. Each grid interval represents 1 μm.

Supplementary Movie 4. Time-lapse imaging of cell proliferation during SynTrogo. Ligand-expressing HEK293T cells were coincubated with receptorexpressing HeLa cells. Fluorescent images were captured every 3 min for 18 h. Numbers indicate hours:minutes:seconds. Scale bar, 20 μm.

Supplementary Movie 5. Time-lapse imaging of SynTrogo in HEK293T– astrocyte interaction. Ligand-expressing HEK293T cells were coincubated with receptorexpressing astrocytes. Fluorescent images were captured every 20 s. Numbers indicate hours:minutes:seconds. Scale bar, 20 μm.

Supplementary Movie 6. CLEM-based identification and 3D reconstruction of an axon in control. Confocal images show GFP-positive axons (green) and RFP-labeled astrocytes (red) in the hippocampal CA1 region. Fluorescence signals were used to identify the corresponding structures in serial EM sections. The yellow contour indicates the axon– astrocyte interface. The final 3D reconstruction illustrates the spatial relationship among the axon (green), dendritic spines (orange), and synaptic vesicles (red dots). Scale bars, 20 μm (confocal) and 1 μm (EM).

Supplementary Movie 7. CLEM-based identification and 3D reconstruction of an axon under SynTrogo conditions. Confocal images show colocalized GFP (ligand) and RFP (receptor) signals used to select regions for EM correlation. Serial EM sections reveal a membrane-enwrapped axonal profile within an astrocytic process (red), with the axon–astrocyte interface outlined in yellow. The 3D reconstruction depicts the enwrapped axonal segment together with dendritic spines (orange) and synaptic vesicles (red dots). Scale bars, 20 μm (confocal) and 1 μm (EM).

Supplementary Data 1. The oligonucleotide sequences used for plasmid construction in this study. The table includes the construct name, forward and reverse primer sequence (5'–3′).
